# Supplementary material for: Use of pharmacy services in community-dwelling middle-aged and older adults; findings from The Irish Longitudinal Study on Ageing (TILDA)
Source: Explor Res Clin Soc Pharm. 2023 Apr 13;10:100265. doi: 10.1016/j.rcsop.2023.100265 (PMC10173775; doi:10.1016/j.rcsop.2023.100265)

**Use of pharmacy services in community-dwelling middle-aged and older adults; findings from The Irish Longitudinal Study on Ageing (TILDA)**

*Logan T. Murry^1^, Michelle Flood^1^, Alice Holton^1^, Rose Anne Kenny^2^, Frank Moriarty^1,2^*

**Appendices**

**Appendix 1.** TILDA CAPI Pharmacy Service Questionnaire Item

In the last 12 months when [you/name] visited the pharmacy did [you/he/she] avail of any of the following services?

1. Request advice about medications

2. Blood pressure monitoring

3. Smoking cessation advice

4. Weight management advice

5. Diabetes risk assessment

6. Asthma control testing

7. Allergy testing

8. Cholesterol checks

9. Vaccination

10. Did not visit pharmacy in the last 12 months

95. Other (please specify) [Go to HU081oth]

96. None of these services

98. DK

99. RF

If Other: (please specify)

**Appendix 2.** Relationship between high risk medication and patient requesting advice about medication at a community pharmacy, frequencies and chi-square test p-values.

| High Risk Medication Category | Total  n (%)  (n = 5782) | Did not Request Advice About Medications  n (%)  (n=4459)​ | Requested Advice About Medications  n (%)  (n=1323)​ | (p-value) |
| --- | --- | --- | --- | --- |
| **Anticoagulant** | 358 (6.2) | 293 (6.0) | 65 (8.0)​ | 0.009 |
| **Antiplatelet** | 1,368 (23.7) | 1,127 (22.6) | 241 (30.7) | <0.001 |
| **Diuretic** | 725 (12.5) | 611 (12.2) | 114 (14.5) | 0.074 |
| **Hypoglycemic** | 417 (7.2) | 357 (7.1) | 60 (7.6) | 0.623 |
| **Insulin3** | 83 (1.4) | 67 (1.34) | 16 (2.0) | 0.128 |
| **NSAID** | 317 (5.5) | 250 (5.0) | 67 (8.5) | <0.001 |
| **Opioid** | 263 (4.5) | 217 (4.3) | 46 (5.9) | 0.059 |
| **Any High Risk Medication** | 2,444 (42.3) | 2,036 (40.7) | 408 (51.9) | <0.001 |

**Appendix 3**. Factors associated with reporting requesting advice about medications in multivariate logistic regression.


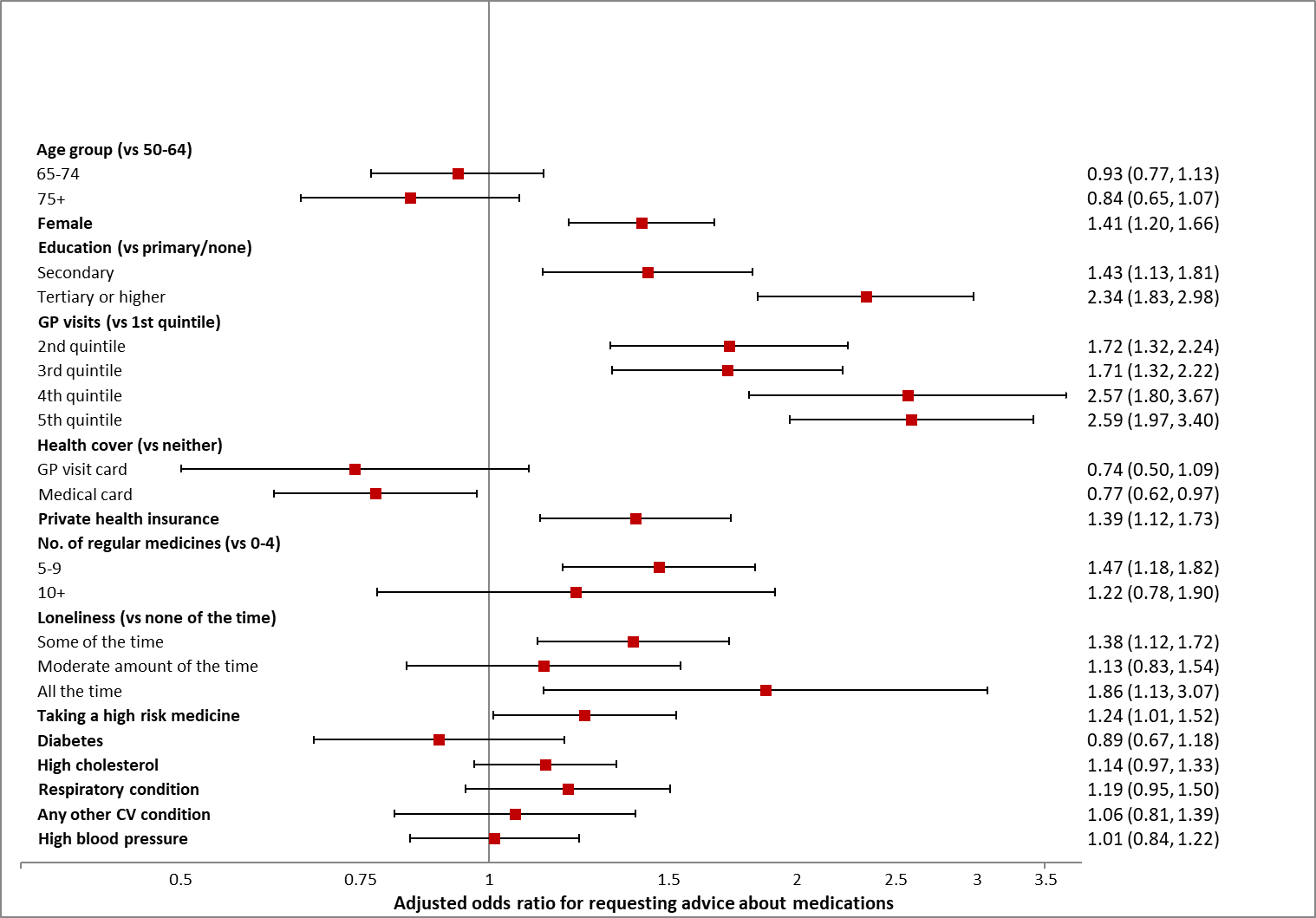

Supplement: Supplementary file 1 — Supplementary material [file mmc1.docx]
